# Tensor decomposition reveals discrete cortical reorganization under graded propofol anesthesia

**DOI:** 10.3389/fnins.2026.1950745

**Published:** 2026-10-02

**Authors:** Chuan-Di Tong, Yun Wang, Zhou-Jing Yang, Jie-Lu Yu, Jie-Chun Huang, Dong-Li Li, Meng-Fan Hu, Chen Li, Ruo-Yu Xia, Xue-Ying Li, Yi-Qi Wu, Ruo-Ying Xie

**Affiliations:** 1 Department of Anesthesiology, Huashan Hospital, Fudan University, Shanghai, China; 2 Department of Cardiothoracic Surgery, Huashan Hospital, Fudan University, Shanghai, China; 3 Department of Otolaryngology-Head and Neck Surgery, Shanghai Ninth People’s Hospital, Shanghai Jiaotong University School of Medicine, Shanghai, China; 4 Institute of Neuroscience, Key Laboratory of Primate Neurobiology, CAS Center for Excellence in Brain Science and Intelligence Technology, Chinese Academy of Sciences, Shanghai, China

**Keywords:** bicoherence, electroencephalography, general anesthesia, propofol, tensor decomposition

## Abstract

**Background:**

Although propofol-induced anesthesia is known to suppress consciousness by promoting low-frequency frontal oscillations through thalamocortical circuits, a comprehensive understanding of its whole-brain coupling feature set remains elusive. Specifically, the global reorganization of nonlinear cross-frequency coupling remains poorly characterized due to analytical limitations.

**Methods:**

We analyzed 64-channel EEG data from 17 participants across five states (awake and target propofol concentrations of 1, 2, 3, and 4 mcg/ml, defined as States 1 to 5). After computing time-frequency representation (TFR) and bicoherence, Parallel Factor Analysis (PARAFAC) was used to extract unbiased spatio-spectral components from both linear and nonlinear measures.

**Results:**

PARAFAC decomposition isolated distinct linear and nonlinear neurodynamic components driving the anesthetic transition. While linear spectral features and signal complexity transitioned in a gradual, monotonic manner. PARAFAC tensor decomposition of high-dimensional whole-brain bicoherence identified two robust, intrinsically decoupled nonlinear components with distinct spatial-spectral profiles. Model comparison demonstrated that frontal slow-wave self-coupling (Component 1) undergoes a discrete, stepwise state-space reconfiguration during loss of consciousness, whereas posterior alpha coupling (Component 2) exhibits a smooth, continuous decay with propofol titration. Phase-space kinetic trajectories confirmed a highly directed transition at unconsciousness onset, followed by confined local oscillations during deep anesthesia.

**Conclusion:**

This multi-dimensional decomposition framework captures discrete versus continuous neural dynamics, providing objective, state-sensitive biomarkers for precisely monitoring anesthesia depth and cortical state transitions.

## 1 Introduction

Decoding the neural mechanisms behind consciousness remains one of the greatest challenges in human neuroscience (Tononi, 2004). Despite decades of dedicated research, controversy still outweighs consensus (Bayne et al., 2016). One of the most effective approaches to investigating consciousness and arousal involves comparing the individual physiological characteristics of the conscious waking state with general anesthesia (Lee et al., 2013; Hagihira, 2015; Koch et al., 2016; Malekmohammadi et al., 2018; Nani et al., 2019), the most common non-pathological state of unconsciousness (Purdon et al., 2013; Manohara et al., 2025). Profiling the similarities and differences between these distinct states using non-linear parameters—and mapping their corresponding regional involvement in the human brain—is essential for a profound understanding of consciousness and arousal.

Approximation entropy (ApEn) (Bruhn et al., 2000), spectral entropy (SE) (Ajayan et al., 2023), and the recently introduced permutation entropy (PE) (Liang et al., 2015) have all been found to correlate with changes in anesthetic depth, further establishing the role of nonlinear EEG generation mechanisms in anesthesia. Other nonlinear synchrony measures, such as bicoherence, can capture phase coupling between oscillations at different frequencies, thereby reflecting the strength of thalamocortical resonance (Abe et al., 2024). Multiple studies have utilized bicoherence analysis of the EEG to explore characteristic changes during drug-induced transitions in consciousness. For instance, Teiji et al. (Hayashi et al., 2014) found that the nonlinear regulation of the occipital EEG differs from the frontal EEG across different stage of anesthesia; concurrently, the combined use of dexmedetomidine and sevoflurane was observed to increase both alpha and theta bicoherence (Zhang et al., 2022), and bicoherence can also serve as a tool to evaluate the analgesic effect during surgery (Uno et al., 2024). However, existing literature is primarily restricted to investigating bicoherence solely within the frontal lobe or other local regions, leading only to conclusions about local nonlinear changes, and consequently failing to establish macroscopic neurodynamic mechanisms regarding “whole-brain” (Hayashi et al., 2008; Akeju et al., 2014). This limitation hinders our understanding of the necessity of quantifying cross-regional, cross-frequency nonlinear interactions at the whole-brain scale. Quantifying such higher-order interactions helps reveal the functional consequences of this resonance (e.g., the integrity of information processing in large-scale networks).

While the macroscopic electrophysiological signatures of propofol-induced anesthesia—most notably the augmentation of frontal alpha oscillations—are well-documented, a systematic mapping of how whole-brain nonlinear phase coupling reorganizes across graded anesthetic states remains elusive. This study addresses a fundamental question: does the trajectory of brain dynamics during graded propofol induction follow a globally uniform, linear progression, or is it characterized by hidden, discrete nonlinear reorganizations? To resolve this, we recorded high-density EEG from 17 participants across five dose-defined recording conditions (following graded propofol concentrations) and implemented a multidimensional tensor-based analytical framework. We integrated time-frequency representations (TFR) to assess linear amplitude dynamics with bicoherence to quantify nonlinear cross-frequency coupling. By applying Parallel Factor Analysis (PARAFAC) (Xiao et al., 2021) for the unbiased extraction of spatio-spectral components, we tracked their evolution across brain states. Our findings demonstrate that while linear power shifts monotonically, nonlinear coupling components undergo a distinct, discrete reorganization. During the transition state between light sedation and deep anesthesia.

## 2 Methods

### 2.1 Participants

EEG data were gathered at Huashan Hospital, Fudan University (Shanghai, China). We included patients who were aged 18 to 65 years, had an American Society of Anesthesiologists physical status classification of I, II, and were scheduled for general surgery under general anesthesia. The Consolidated Standards of Reporting Trials (CONSORT) diagram is shown in Supplementary Figure S1. Exclusion criteria were as follows: history of an allergic response to propofol, history of neurological or psychological disease, history of severe cardiovascular disease, history of metabolic disease, and any anticipate a difficult airway. All patients fasted for 6–8 h prior to surgery without receiving any premedication. Patient characteristics and variables was shown in Supplementary Table S1.

### 2.2 Study procedure

We collected EEG data from 17 patients undergoing propofol-induced gradient to unconsciousness during induction of anesthesia and obtained their informed consent (Supplementary Figure S1). The EEG electrode cap was set up during the awake state and before the raw EEG signals were recorded. All subjects were asked to lie quietly on the operating table for several minutes, then close their eyes for 3 min before recording. Then propofol was administered intravenously via a micro-pump with targeted controlled infusion (TCI, Schnider model, Medcaptain, Medical Technology Co., Ltd.). During the ascending propofol induction sequence, EEG recordings were obtained under five dose-defined recording conditions, designated State 1–State 5. These labels represent experimental recording conditions defined by the target propofol effect-site concentration. The level of consciousness was assessed using the Observer Assessment of Alertness/Sedation (OAA/S) scale. Each patient received propofol via TCI at four increasing target effect-site concentrations (1 mcg/ml, 2 mcg/ml, 3 mcg/ml, 4 mcg/ml). Normal saline was then infused at a rate of 4 mL kg ^−1^ h^−1^. State 1 corresponded to the awake baseline condition before propofol administration (target concentration: 0 mcg/ml), whereas States 2–5 corresponded to target effect-site concentrations of 1, 2, 3, and 4 mcg/ml, respectively. At each target concentration, after the effect-site concentration had reached a stable level, a continuous 5–7 min resting-state EEG segment was recorded. Behavioral responsiveness was assessed immediately after each EEG recording window using the Observer’s Assessment of Alertness/Sedation (OAA/S) scale to avoid motor or auditory contamination during resting-state EEG acquisition. Target concentrations of 1–2 mcg/ml generally corresponded to sedation/light anesthesia (OAA/S scores of 3–4), whereas concentrations of 3–4 mcg/ml were associated with profound unresponsiveness (OAA/S scores of 0–1). Individual OAA/S scores at each recording condition and the distribution of loss of responsiveness (LOR, defined as OAA/S ≤ 2) across target concentrations are provided in [Supplementary Table S2/Supplementary Figure S2].

The vital signs of the subjects were continuously monitored. After the EEG recording was complete, analgesic and neuromuscular blocking agents were administered for tracheal intubation, followed by general anesthesia for the scheduled surgery.

### 2.3 EEG pre-processing

We processed and analyzed all EEG signals using MATLAB 2020b (The MathWorks Inc., Massachusetts) and the EEGLAB toolbox. The EEG data were first down-sampled to 500 Hz using the pop_resample.m function. A band-pass filter (0.1–45 Hz) was applied via the pop_eegfiltnew.m function to reduce interference from electromyographic activity and baseline drift. Additionally, a 50 Hz notch filter was implemented to mitigate electrical noise, which can substantially distort the signal and impede accurate analysis of neural or physiological activity. In addition to the removal of bad channels identified by visual inspection, data segments with noticeable noise contamination were also excluded manually. For each participant, Independent Component Analysis (ICA) using the Infomax algorithm was conducted to remove artifacts related to eye blinks and facial electromyography.

### 2.4 Time-frequency analysis

The continuous EEG data were segmented into 3-s epochs with 50% overlap using the FieldTrip toolbox. Time-frequency representations (TFRs) were computed for each epoch using a multitaper method (Hanning window) in the frequency range of 0.5–50 Hz, discretized into 89 frequency bins. To ensure data quality, a variance-based filtering step was applied: for each subject and state, the overall variance across all channels, frequencies, and time points was computed, and the 150 most stable epochs (with the lowest variance) were selected for subsequent analysis. Finally, the TFRs were decomposed into six standard frequency bands: Delta (0.5–4 Hz), Theta (4–8 Hz), Alpha (8–12 Hz), Low Beta (12–15 Hz), High Beta (15–25 Hz), and Gamma (25–40 Hz) (Brown et al., 2010). The band-specific power was averaged across subjects and time, and visualized as scalp topographies.

### 2.5 Dimensionality analysis

Dimensionality analysis was performed on each 3-s EEG segment using both the original signals and signals filtered into six standard frequency bands. For each segment (channels × time points) in the broadband and band-limited signals, the channel-wise covariance matrix C was computed. Eigenvalue decomposition was then applied to C to obtain eigenvalues (*λ*₁, *λ*₂,…, *λ*n) using eig function in MATLAB, representing the variance accounted for by each orthogonal component in the channel space. The effective dimensionality was estimated using the participation ratio method, defined as:

$$\dim(C)=\frac{{\left(\sum_{i=1}^{n}\lambda_{i}\right)}^{2}}{\sum_{i=1}^{n}{\lambda_{i}}^{2}}$$

This metric quantifies the number of dominant, independent neural sources contributing to the EEG signal, with values ranging from 1 (indicating perfect correlation among channels) to *n* (reflecting fully independent channels).

### 2.6 Entropy analysis(ApEn)

To quantify signal regularity, we computed Approximate Entropy (ApEn) on 3-s epochs derived from each state. The ApEn algorithm requires the specification of two parameters: the embedding dimension (*m*) and the tolerance (*r*). In our analysis, m was set to 2 to capture short-range patterns, and r was defined as 0.2 multiplied by the standard deviation of the epoch, ensuring the similarity criterion was adapted to the local signal variance.

Raw EEG data were first filtered (0.5–40 Hz) to remove artifacts and slow drifts. The data were then segmented into 3-s epochs with 50% overlap and analyzed using the *Entropy_measures* toolbox in MATLAB.

Approximate entropy (ApEn):

$$\text{ApEn}(m,r,N)=\emptyset^{m}(r)-\emptyset^{m+1}(r)$$

$$\emptyset^{m}(r)=\frac{1}{N-m}\sum_{i=1}^{N-m}\ln C_{i}^{m}(r)$$

The embedding dimension was set to *m* = 2.

### 2.7 Parallel factor analysis (PARAFAC)

To extract robust, physiologically interpretable spatiospectral patterns of cortical nonlinear coupling and linear oscillatory dynamics across graded anesthetic states, third-order PARAFAC tensor decomposition was separately performed on whole-brain wavelet bicoherence and TFR datasets. All tensor modeling and decomposition procedures were implemented in MATLAB R2020a using the standard PARAFAC solver from the N-way toolbox (Andersson and Bro, 2000). Two structurally consistent but physiologically specialized third-order tensors were constructed to integrate multi-dimensional electrophysiological features with unified spatial and experimental modes while retaining distinct spectral-temporal dimensions.

For the bicoherence tensor, the three orthogonal modes were defined as follows: (1) a joint spatial-subject mode integrating scalp EEG channels and individual participants to capture population-level topographical coupling patterns; (2) a bispectral coupling mode consisting of vectorized frequency-by-frequency ( f1×f2 ) pairwise combinations, encoding full-band quadratic phase coupling profiles; and (3) an experimental condition mode corresponding to the five graded anesthetic states.

For the TFR tensor, the model adopted identical joint spatial-subject and experimental condition modes to ensure direct cross-modal comparability with the bicoherence decomposition. Critically, its second mode differed fundamentally and integrated frequency bins and sequential trial time segments (frequency–time plane), explicitly characterizing the dynamic evolution of linear oscillatory power across spectral and temporal domains during anesthesia transition. Prior to decomposition, unified normalization was applied to each full tensor dataset to eliminate inter-subject and inter-state amplitude bias and ensure equitable pattern decomposition across experimental conditions. The PARAFAC model decomposes the original high-dimensional tensor into a linear combination of low-rank trilinear components, mathematically formulated as:

$$x_{\mathrm{ijk}}=\sum_{r=1}^{R}a_{\mathrm{ir}}b_{\mathrm{jk}}c_{\mathrm{kr}}+e_{\mathrm{ijk}}$$

Where *R* indicates the number of latent components; a , b and c denote the loading matrices corresponding to the three modes of each tensor, respectively; and $e_{\mathrm{ijk}}$ represents the residual reconstruction error. Strictly consistent with our computational configuration, non-negativity constraints were imposed on all three tensor modes for both datasets to conform to the non-negative mathematical and physiological properties of bicoherence coupling strength and spectral power. Initialization employed the Direct Tri-Linear Decomposition (DTLD) method, which has been formally demonstrated to yield superior parameter estimation accuracy (Onken et al., 2016). The iterative optimization was terminated when the relative variation of the residual sum of squares fell below a convergence threshold of $\;1\times 10^{-6}$ , with a maximum of 2,500 iterations predefined to prevent infinite iteration. Default tensor scaling was adopted to concentrate component variance within the spatial mode, and fitting residuals were recorded every ten iterations to dynamically monitor model convergence. The optimal component number for each dataset was determined using the core consistency diagnostic (CORCONDIA) criterion (Wang et al., 2018). Which quantitatively evaluates the trilinear structural conformity of the decomposed tensor by comparing the reconstructed core tensor with the ideal diagonal core structure, with higher core consistency values indicating more parsimonious and reliable model solutions. Model fitness was quantified as the explained variance ratio, calculated based on the total sum of squares and residual sum of squares of the original tensor. After systematic rank screening across candidate component numbers, the TFR time-frequency tensor adopted a 3-component optimal solution, and the bicoherence coupling tensor adopted a 2-component optimal solution.

### 2.8 Bicoherence analysis

Wavelet-based bicoherence analysis was employed to characterize nonlinear phase coupling in cortical EEG signals across anesthetic states. Continuous EEG recordings were segmented into 7-s epochs with 50% overlap; at a sampling rate of 500 Hz, each epoch comprised 3,500 data points. For each epoch, complex Morlet wavelet transform was applied to derive time-frequency representations of the signal across a frequency range of 1 to 20 Hz, with 50 linearly spaced frequency bins. An adaptive wavelet cycle scheme was adopted: the number of cycles increased linearly from 3 at the lowest frequency to 7 at the highest frequency, which reduces edge artifacts in the low-frequency band while maintaining adequate frequency resolution in the high-frequency band. Bicoherence values for each frequency pair $\left(f_{1},f_{2}\right)$ were calculated from the wavelet coefficients to quantify the strength of quadratic phase coupling between frequency components $f_{1},f_{2}$ , and their sum frequency $f_{1}+f_{2}$ , with values bounded between 0 (no phase coupling) and 1 (perfect phase coupling).

To evaluate the statistical significance of the estimated coupling and identify spurious coupling driven by random signal fluctuations, a surrogate data procedure was performed: 100 surrogate time series were generated via phase randomization with preserved amplitude spectra, and bicoherence was recomputed for each surrogate to construct a null distribution. Significance was determined at the α = 0.05 level for statistical interpretation of results. The raw bicoherence matrices of all channels and epochs were used as input for subsequent tensor decomposition analysis.

### 2.9 Dynamic trajectory analysis

Time-resolved projection weights were computed via a continuous sliding time window approach, constructing evolution trajectories in the W_1_–W_2_ phase plane for each subject across the four state transition stages. Group-averaged trajectories were derived by averaging weight values across all subjects at each corresponding time point. Three dynamic metrics were calculated to quantitatively characterize trajectory patterns: instantaneous speed, net displacement, and trajectory directness. Specifically, instantaneous speed was defined as the Euclidean distance between consecutive time points on the trajectory, reflecting the magnitude of weight change per time step within each transition stage. Net displacement corresponded to the Euclidean distance between the start and end points of stage-specific trajectories, quantifying the overall magnitude of neural state transition in the phase space. Trajectory directness was calculated as the ratio of net displacement to total trajectory path length, ranging from 0 to 1; lower values indicate trajectories dominated by localized reciprocal oscillation, while higher values represent more directed, linear neural state migration.

To statistically dissociate discrete abrupt state switching from smooth continuous neural drift at the individual level, two competitive temporal models were independently fitted to each participant’s full time-course weight series. The piecewise stepwise model adopted task-stage-aligned fixed breakpoints to capture discontinuous state transitions, while the fourth-order global polynomial model was used to characterize uninterrupted smooth temporal variation. To eliminate numerical ill-conditioning of high-order polynomial regression, all time indices were standardized via mean centering and z-score normalization prior to model fitting.

Model fitting performance was comprehensively evaluated using degree-of-freedom corrected Akaike Information Criterion (AIC), coefficient of determination (*R*^2^), and root-mean-square error (RMSE) for unbiased and fair cross-model comparison. Given that raw AIC values are inherently scaled by data length and lack standalone interpretability, only within-subject relative model differences were used to determine dynamical preference. Individual-level model discrepancy was quantified as ΔAIC (ΔAIC = AIC_stepwise − AIC_continuous), with negative values indicating superior fitting of the discrete stepwise model. Full model construction parameters, mathematical formulas, fitting pipelines, and individual judgment criteria are provided in Supplementary method 1.

### 2.10 Statistical analysis

Our statistical analyses encompassed multiple metrics including EEG spectral power, broadband TFR, six-sub-band TFR, as well as PARAFAC-derived projection weights and phase-plane dynamic metrics. Topographic maps of both broadband and sub-band EEG power were generated to visualize the spatial distribution of neural oscillatory activity across different states.

Normality tests were first conducted on the differences between states; based on these results, either the Wilcoxon signed-rank test (for non-normally distributed parameters) or paired t-test (for normally distributed parameters) was applied. For the six-sub-band TFR data, a three-way ANOVA was employed to assess interactions among frequency bands, brain regions, and experimental conditions, with post-hoc comparisons conducted using Bonferroni correction for multiple comparisons. For steady-state projection weights extracted from PARAFAC decomposition, Friedman nonparametric repeated measures tests were used to evaluate overall differences across the five anesthesia states, as the data did not meet the normality assumption required for parametric analysis of variance. Post-hoc pairwise comparisons were performed using Dunn’s test with correction for multiple comparisons to identify significant differences between individual state pairs.

All reported *p*-values were adjusted for multiple testing using appropriate correction methods. Results are reported as medians (25th–75th percentiles) or means ± standard deviation, as appropriate. Mean/median differences and 95% confidence intervals between groups were estimated using bootstrap methods. A threshold of *p* < 0.05 (corrected) was adopted for statistical significance. All hierarchical data aggregation rules, pseudo-replication control strategies, and detailed correction specifications are documented in Supplementary method.

## 3 Results

### 3.1 Propofol-induced cortical simplification and dimensionality reduction

To establish the fundamental evolution of electrophysiological states during propofol induction, we first analyzed high-density EEG data from 17 participants across five graded levels of propofol concentrations (State 1: awake baseline; States 2–5: incremental propofol concentrations of 1–4 mcg/ml; Figure 1A). Time-frequency representations (Figure 1B) and topographical maps (Figure 1C) revealed a progressive spectral evolution coupled with deepening anesthesia.

**Figure 1:**
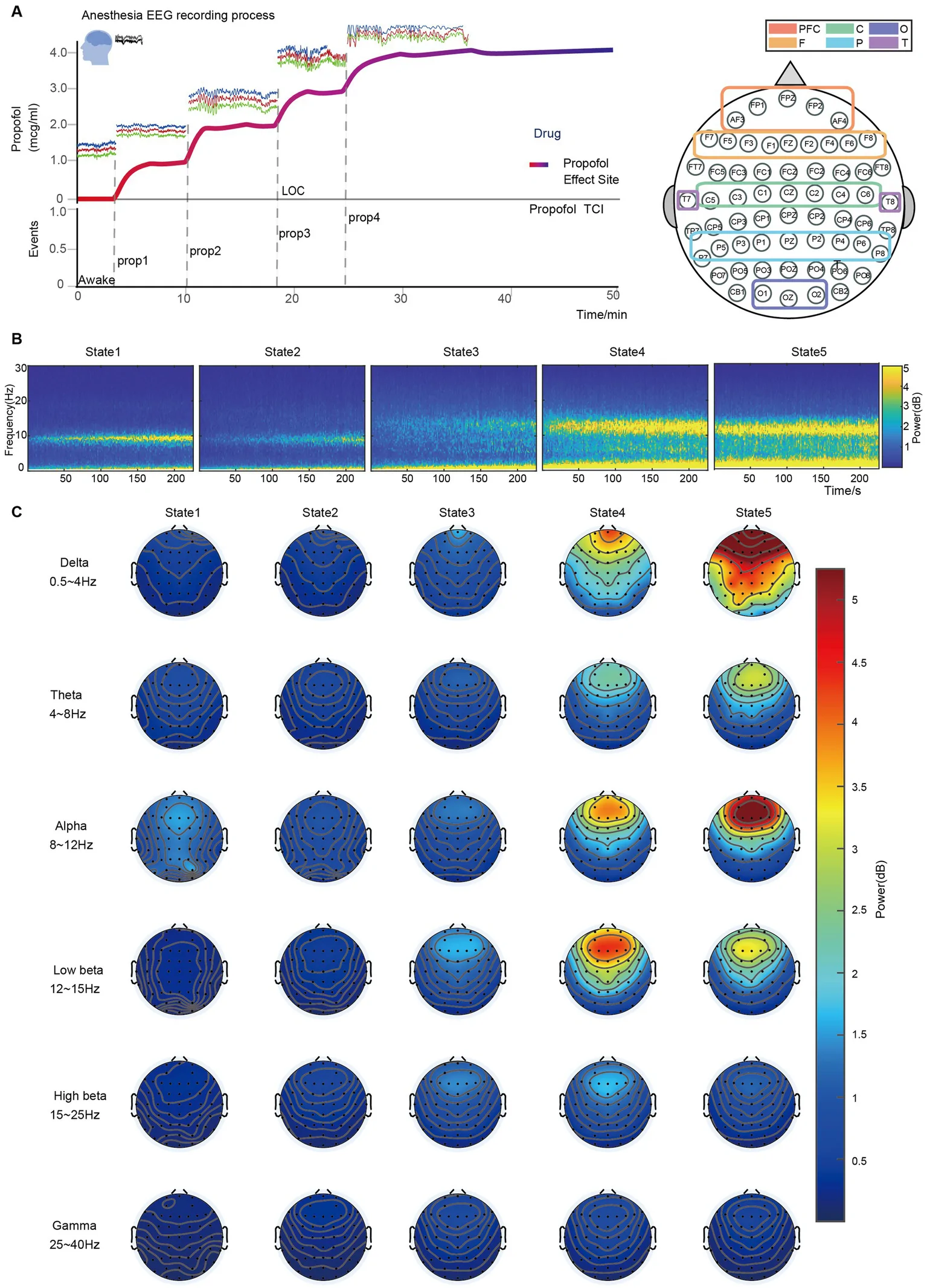
EEG recording paradigm and spectral-spatial features across propofol concentrations. **(A)** Left: Schematic of the experimental protocol. Resting-state EEG data were recorded sequentially from each participant during wakefulness and at four increasing target effect-site concentrations of propofol (P1–P4, corresponding to 1, 2, 3, and 4 mcg/ml, respectively). Each recording began after the effect-site concentration had stabilized for several minutes. Right: Electrodes grouped by anatomical regions of interest (prefrontal, frontal, central, parietal, and occipital) are outlined in different colors, corresponding to regions used in subsequent region-based spectral analyses. **(B)** The group median spectrograms across the five states from all 17 participants. **(C)** Scalp topographic maps showing group-averaged EEG power across five frequency bands-delta (0.5–4 Hz), alpha (8–12 Hz), low beta (12–15 Hz), high beta (15–25 Hz), and gamma (25–45 Hz)-under each state. The color bar indicates the magnitude of spectral power, expressed in decibels (dB). Warmer colors represent higher power levels.

During the initial induction phases (States 2 and 3), we observed a transient surge in beta (12–25 Hz) and gamma (25–40 Hz) activity, followed by the emergence of robust frontal alpha (8–12 Hz) oscillations and significantly increased central delta (0.5–4 Hz) power relative to baseline. In deeper anesthesia (States 4 and 5), alpha oscillations expanded across the fronto-central regions, accompanied by widespread delta elevations and the marked suppression of high-frequency (beta/gamma) activity, reflecting a classic “low-frequency enhancement and high-frequency suppression” pattern (Figure 2A). These findings provide a robust macroscopic foundation for subsequent nonlinear dynamical analyses.

**Figure 2:**
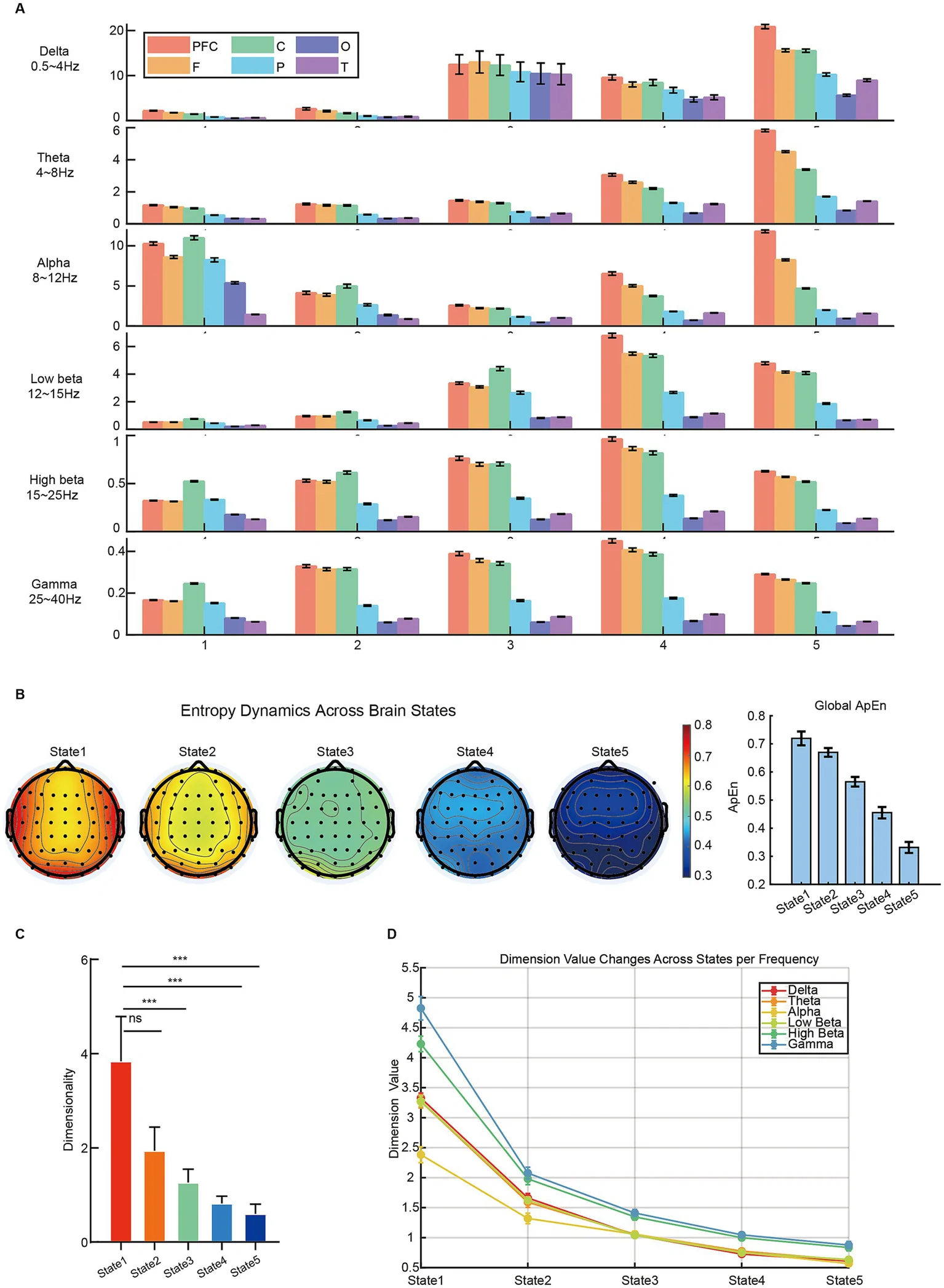
Propofol-induced cortical simplification and dimensionality contraction. **(A)** Power spectral density changes across six frequency bands in brain regions under five states. Regions are color-coded (prefrontal, frontal, central, parietal, temporal, occipital), and values represent group-averaged spectral power (dB), N = 17 participants. **(B)** Approximate entropy (ApEn) topographies across the five states, illustrating spatial patterns of EEG complexity. Color bar indicates normalized ApEn. The color scale represents the approximate entropy (ApEn) values across EEG electrode locations, with warmer colors indicating higher ApEn values and cooler colors indicating lower ApEn values. Right panel displays global ApEn values, showing a monotonic decrease in signal complexity as anesthesia depth deepened (pair-*t* test, ***p < 0.001). Error bars represent mean ± standard error of the mean (SEM) across N = 17 participants. **(C)** Global dimensionality of EEG signals across the five states. (Rank-sum test, ***p < 0.001). **(D)** Frequency-specific analysis of EEG dimensionality across the five states. Dimensionality decreased across all frequency bands as propofol-induced anesthesia depth deepened.

However, traditional spectral analysis primarily reflects linear amplitude changes and remains insufficient to characterize the underlying nonlinear regularity or coordinate structure of neural signals. To address this, we first employed Approximate Entropy (ApEn) (Liang et al., 2015) to evaluate the regularity of the recorded EEG dynamics across anesthetic conditions. All trial epochs were averaged within each participant prior to statistical testing, establishing the individual participant (*N* = 17) as the sole independent sampling unit. A non-parametric Friedman test across the five anesthetic states revealed a significant global decline in brain complexity ( $\chi^{2}$ (4) = 64.12, ***p < 0.001). Pairwise post-hoc comparisons using Wilcoxon signed-rank paired tests with Bonferroni correction demonstrated that ApEn declined monotonically as anesthesia deepened (decreasing from 0.72 ± 0.025 in State 1 to 0.33 ± 0.020 in State 5; pairwise comparisons across successive states, all adjusted ***p < 0.001). Notably, this decline in ApEn was most pronounced over fronto-central scalp regions (Figure 2B), indicating an increase in the regularity and predictability of the recorded scalp EEG signals under propofol.

To further characterize the contraction of the neural state-space from a global perspective and to test whether the observed loss of complexity reflects a simplification of neural coordination, we utilized Singular Value Decomposition (SVD) on participant-aggregated signals to assess the intrinsic dimensionality of the EEG signals. As shown in Figure 2C, the effective dimensionality of the channel covariance matrix contracted significantly as consciousness diminished, paralleling the increase in signal regularity. A global Friedman test confirmed a highly significant main effect of anesthetic state on mean dimensionality ( $\chi^{2}$ (4) = 58.32, ***p < 0.001). Post-hoc pairwise Wilcoxon signed-rank paired tests showed that compared to baseline (State 1), intrinsic dimensionality exhibited no significant change in State 2 (*p* = 0.1615, adjusted), but collapsed dramatically in States 3–5 (*p* = 0.0003 for State 3, ***p < 0.0001 for States 4 and 5; ***p < 0.001). This collapse reflects a reduction in the effective degrees of freedom in spatial covariance across sensors, suggesting a restriction in the diversity of observable scalp-recorded activity patterns during propofol-induced unconsciousness. Finally, to determine whether this dimensionality reduction represents a global systemic shift or is confined to specific oscillatory bands, we evaluated band-specific dimensionality changes across conscious states. Non-parametric repeated-measures analyses across states within each frequency band confirmed significant main effects of anesthetic depth across all six frequency bands (Friedman tests, all ***p < 0.001). All six frequency bands exhibited a consistent downward trend as anesthesia progressed (Figure 2D). These results demonstrate that propofol anesthesia induces not only a macroscopic reorganization of the power spectrum but also a dose-dependent cortical simplification and dimensionality contraction.

### 3.2 PARAFAC tensor decomposition resolves three distinct linear spatio-spectral signatures

While our initial analyses established broad spectral and complexity trends, the overlapping nature of neural oscillations across different brain regions and frequency bands makes it difficult to isolate the distinct neural processes driving the transition to unconsciousness. To disentangle these latent dynamics and comprehensively capture the spatial, spectral, and pharmacological dimensions of the EEG signal, we implemented a PARAFAC (Andersson and Bro, 2000) framework (Figure 3A). Unlike traditional univariate methods, PARAFAC allows for an unbiased, data-driven decomposition of high-dimensional data into stable, interpretable components. We constructed a three-dimensional tensor incorporating anatomical distribution (62 channels × 17 participants), time-frequency information (150 time windows × 89 frequency bins), and five states (Figure 3B). To ensure the reliability of the extracted features, we performed a core consistency diagnostic, which validated a three-component model with a high consistency score exceeding 90%. We further conducted three quantitative stability assessments to numerically verify component reproducibility, including 100 repeated random initializations, leave-one-subject-out cross-validation, and sensitivity tests using alternative epoch screening rules. All three validation pipelines returned median cross-run loading correlation coefficients above 0.98 across spatial, spectral and temporal modes. These three dominant components collectively explained 53.3% of the total variance in the pooled neural activity (Supplementary Figure S3), providing a concise yet robust representation of the anesthesia-induced state-space.

**Figure 3:**
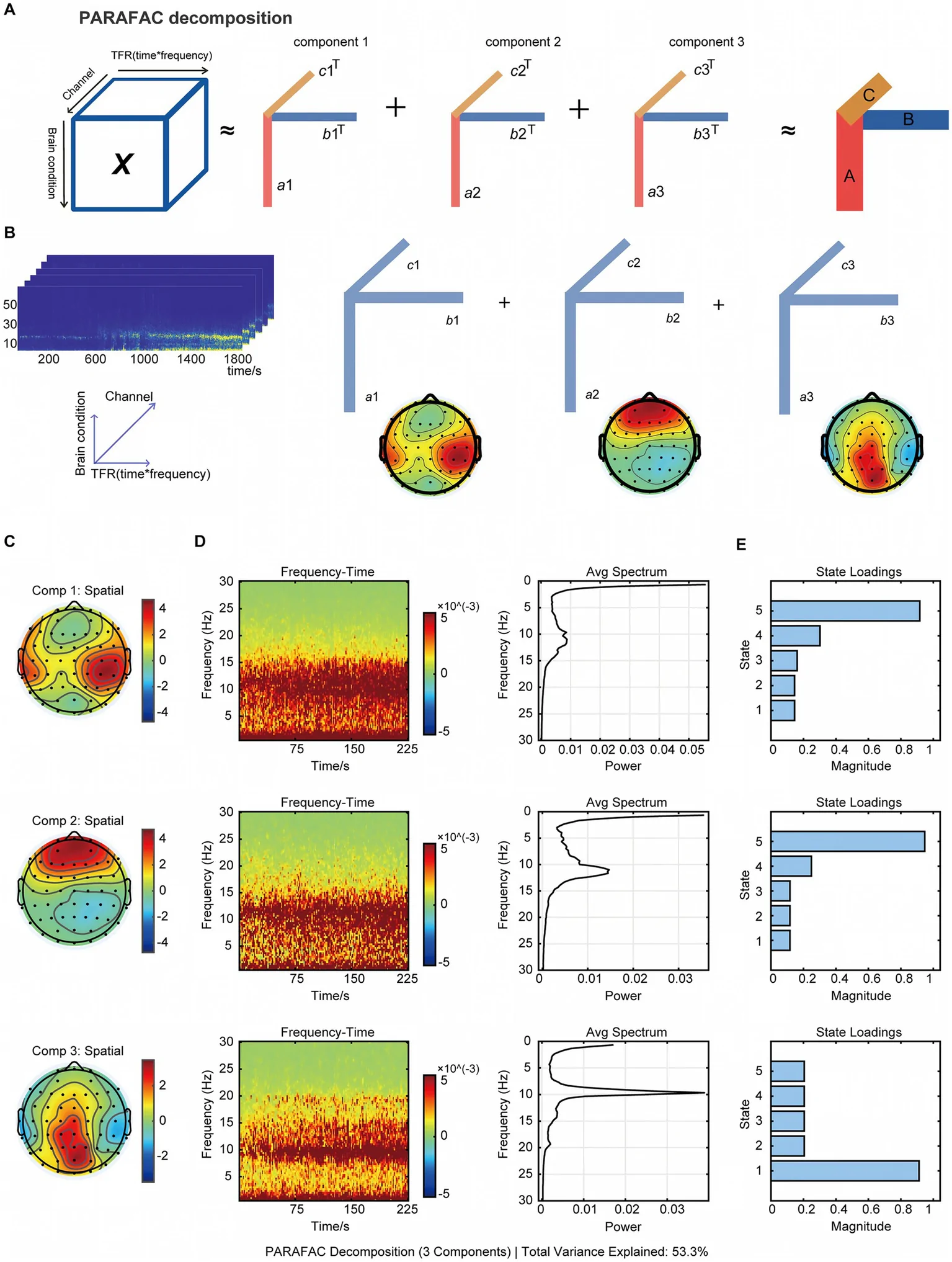
PARAFAC decomposition of linear feature of EEG data across 5 states. **(A)** Schematic of PARAFAC tensor decomposition: A 3 - way data tensor X (dimensions: channel × time - frequency representation (TFR) × time) is decomposed into three components. Each component comprises spatial (an), spectral (bn), and temporal (cn) mode vectors, reconstructing X as a sum of rank - 1 tensors. **(B)** Visualization of component extraction: The stacked tensor of EEG TFRs (left) is decomposed into three components, each associated with a spatial scalp map (colored topographies in C) and corresponding mode vectors. **(C)** The anatomical dimension of the three components in all participants. The color bar represents the activation level of the scalp distribution patterns. The color scale represents the spatial loading values of each PARAFAC component across EEG channels, with warmer colors indicating higher positive loadings and cooler colors indicating lower or negative loadings. **(D)** Frequency-time plots (left) show spectral-temporal dynamics; average spectra (right) depict dominant frequency bands. **(E)** State loadings quantify component contributions across states. Total variance explained by the three components is 53.3%. The spatial mode of the TFR model is unconstrained (constraint vector [1,2,2]), allowing positive and negative loadings to illustrate relative power changes across scalp regions.

The PARAFAC decomposition revealed three neurophysiologically distinct signatures. Component 1 and Component 2 both exhibited a dose-dependent increase in magnitude as propofol concentrations rose (Figure 3E, top and middle), yet they reflected divergent neural mechanisms. Component 1 was characterized by a broad-band low-frequency enhancement (predominantly delta and theta rhythms, <10 Hz) localized over bilateral temporo-parietal areas (Figure 3C,D, top). Component 2 manifested as a focused, narrow-band alpha peak (centered at ~12 Hz) with a clear fronto-central topology (Figure 3C,D, middle). Component 3, in contrast, appears to represent a “signature of wakefulness”. Its intensity was maximal during the awake state and diminished rapidly following anesthetic induction (Figure 3E, bottom). This component was characterized by a spectral peak at the alpha/theta boundary (~9 Hz) and was primarily distributed over the posterior parietal-occipital regions (Figure 3C,D, bottom).

In contrast to traditional univariate analyses that focus on isolated frequency bands or brain regions, our analysis revealed that the transition toward anesthesia is driven by parallel yet functionally complementary processes: the active recruitment of inhibitory slow-wave and frontal-alpha circuits (Components 1 and 2), which occurs in direct temporal opposition to the deconstruction of posterior arousal-maintaining feature (Component 3).

### 3.3 Heterogeneous reorganization of nonlinear cross-frequency coupling

To elucidate whether propofol alters high-order, cross-frequency interactions, we computed the normalized bicoherence across five sequential brain states (Figure 4). Traditional linear analyses capture isolated power shifts, whereas bicoherence tracks quadratic phase coupling (QPC) (Li et al., 2013) to monitor nonlinear network integration(Uno et al., 2024). In the awake baseline (State 1), a prominent nonlinear self-coupling peak was uniformly distributed across all cortical regions, characterized by strong alpha-alpha phase-locking centered along the diagonal at ~10 Hz ( f1=f2 ≈10Hz ; Figure 4A). Upon State 2, this wake-related alpha coupling (Purdon et al., 2015) was markedly suppressed. Crucially, at State 3, the alpha coupling underwent a near-complete dissolution, flatlining near zero across virtually all cortical regions (Figure 4A,B, green traces). During deep anesthesia (States 4–5), the cortex exhibited starkly divergent, spatially heterogeneous trajectories: anterior reconfiguration in the prefrontal and frontal cortices, coupling robustly reemerged as a continuous, cross-frequency zone spanning from (5, 5) Hz to (5, 11) Hz and (11, 11) Hz (Figure 4A). This spatial continuity confirms a unified higher-order coordinated state where low-frequency slow/delta rhythms ( ≈ 5 Hz) nonlinearly modulate alpha rhythms ( ≈ 11 Hz). Concurrently, the single baseline peak (9.76 Hz) bifurcated into a distinct dual-peak architecture at 6.35 Hz and 11.23 Hz (Figure 4B, purple traces). Posterior dissolution: In direct opposition, the parietal and occipital alpha coupling peaks (~12 Hz) were permanently deactivated, flatlining near zero by State 5 and being replaced by generalized low-frequency oscillations (<5 Hz) (Figure 4B; regional classifications and single-channel evaluations are provided in Supplementary Tables S3–S8 and Supplementary Figures S4, S5). In summary, propofol-induced LOC is driven by parallel, spatially opposing nonlinear dynamics: an active cross-frequency reconfiguration (slow-alpha phase coupling) restricted to anterior brain regions, occurring in direct temporal alignment with the systematic phase-decoupling and dissolution of posterior regions.

**Figure 4:**
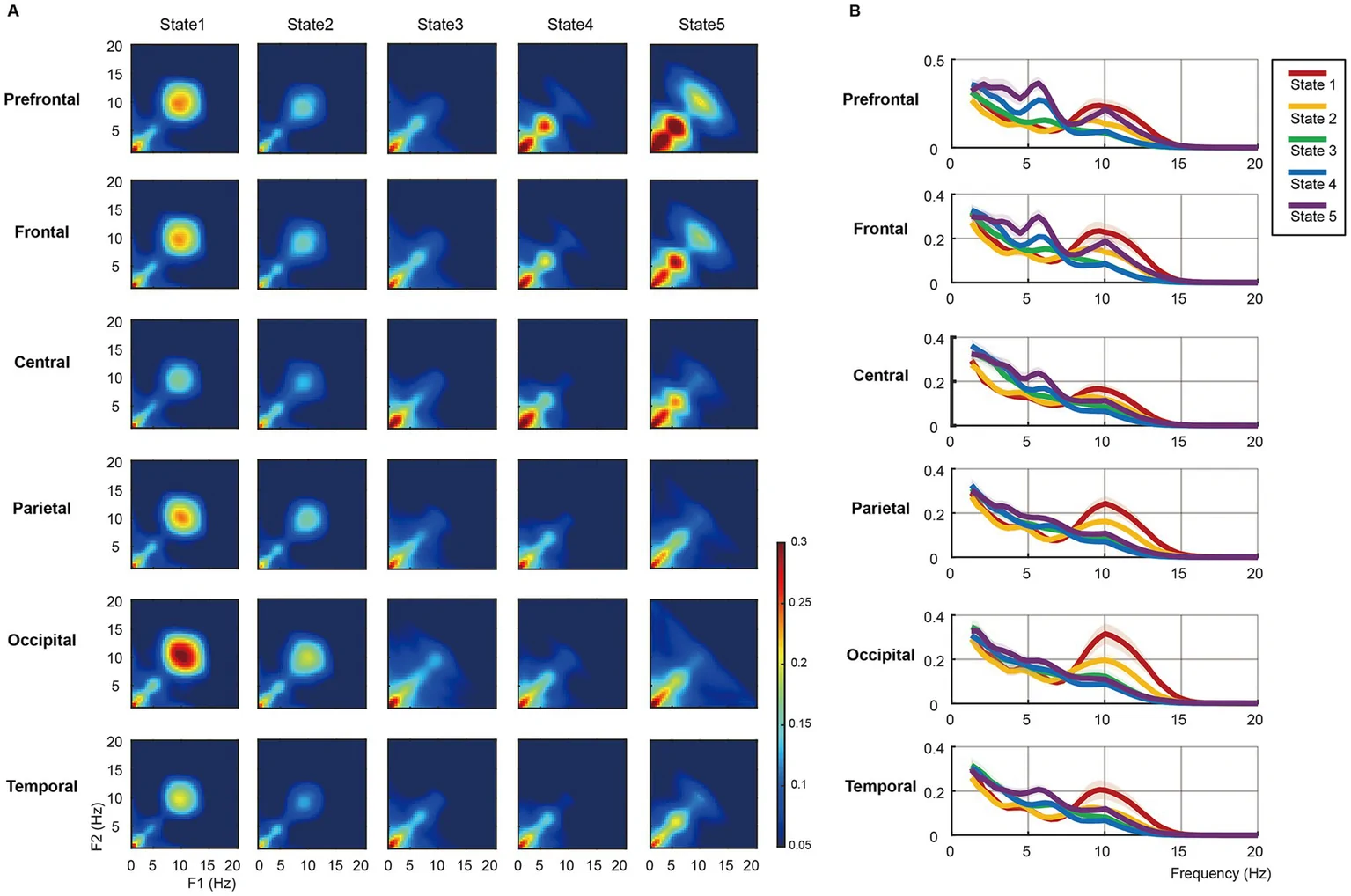
Bicoherence values exhibit region- and frequency-specific modulations across the five states of consciousness. **(A)** Bicoherence spectra (frequency-by-frequency maps) across brain regions and states. Each row represents a different cortical region (prefrontal, frontal, central, parietal, occipital, temporal). Each column corresponds to one of the five consciousness states (from State 1, awake, to State 5, deep propofol anesthesia). Bicoherence values are averaged across subjects. Warm colors indicate higher nonlinear coupling strength; all maps are normalized to the group maximum. **(B)** Diagonal bicoherence values (mean across subjects) as a function of frequency, for each state and region. Diagonal values reflect intra-frequency quadratic phase coupling. Each colored trace represents one consciousness state: red, State 1 (awake); yellow, State 2; green, State 3; blue, State 4; purple, State 5.

### 3.4 Nonlinear EEG dynamics resolve discrete structures in cortical activity

PARAFAC tensor decomposition successfully extracted two intrinsic nonlinear coupling components from high-dimensional bicoherence data, displaying distinct spatial topologies, spectral profiles, and dose-dependent behaviors (Figure 5A). We numerically validated the robustness of this two-component solution via three complementary stability tests: 100 independent random initialization replicates, leave-one-subject-out cross-validation across all 17 participants, and sensitivity analysis adopting alternative trial exclusion thresholds. Median component matching correlations across all spatial, spectral and temporal loadings exceeded 0.97 under all perturbation schemes. Component 1 featured low-frequency self-coupling (<5 Hz) predominantly localized over prefrontal-frontal scalp regions, while Component 2 captured ~10 Hz alpha coupling. Concentrated over posterior occipital sensors. Steady-state projection weights showed prominent non-linear evolutions across the five states (Figure 5B): Component 2 weights monotonically decreased from baseline (S1) to S4 before a slight rebound at S5, whereas Component 1 weights increased with dose and plateaued post-S3. The projection weights of both components altered significantly across states (Component 1: $x^{2}$ (4) = 42.78; Component 2: $x^{2}$ (4) = 44.24; both *p* < 0.0001, Friedman test). Post-hoc pairwise comparisons using Wilcoxon signed-rank paired tests with Bonferroni correction revealed significant differences predominantly between distant states (*p* < 0.05), whereas differences between adjacent states remained non-significant except for Component 2 during the S4 vs. S5 transition (*p* < 0.05). On the W_1_–W_2_ phase plane, the spatial distribution of centroids differed significantly among states (*p* < 0.01, permutation test). Centroids for S1 and S2 were significantly separated from all other states (all *p* < 0.001, *post hoc* Bonferroni correction), while S3–S5 exhibited no significant centroid differences, forming a distinct spatial cluster.

**Figure 5:**
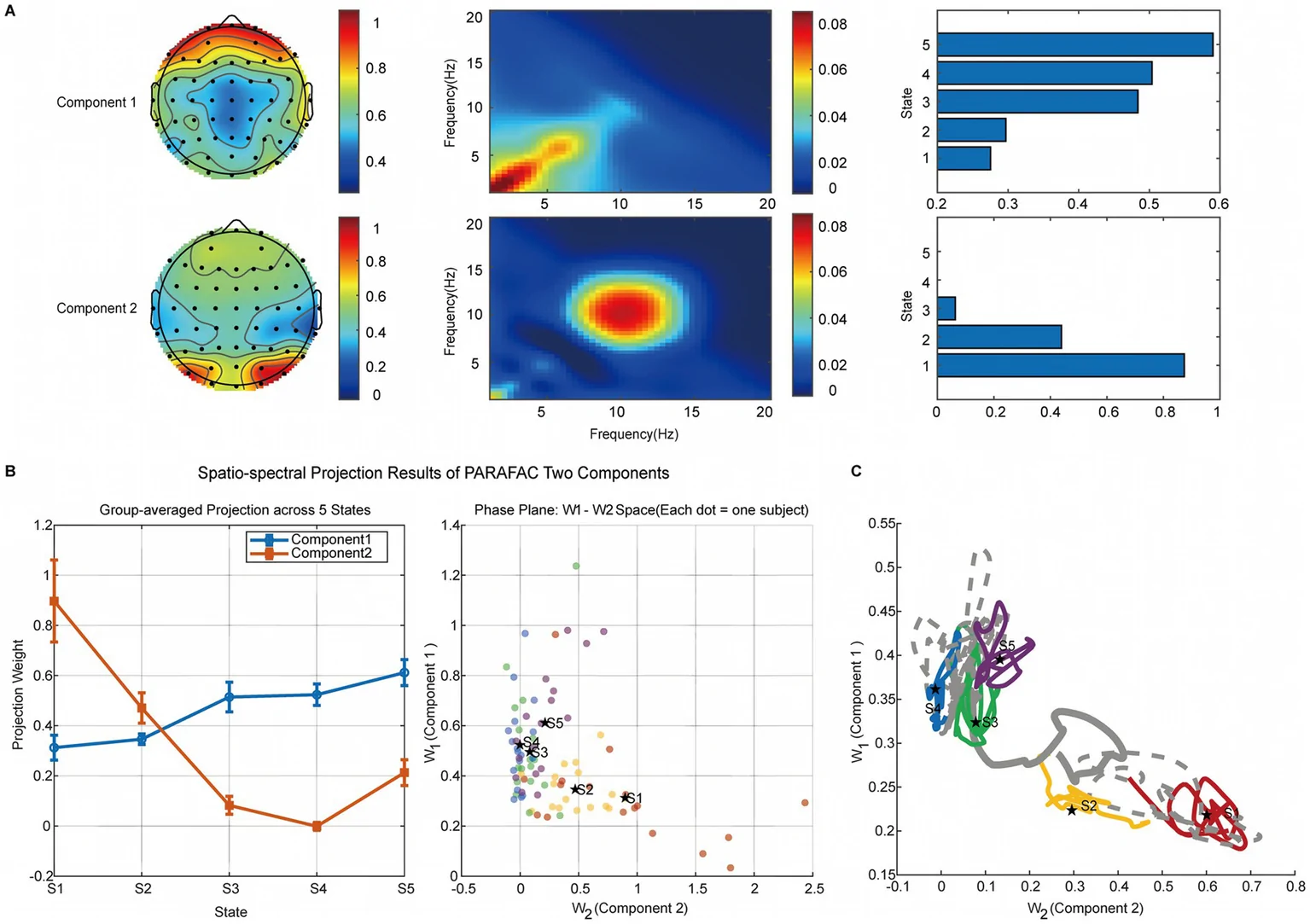
Decomposition and evolutionary profiles of propofol-induced nonlinear coupling. **(A)** Spatial and spectral patterns of the two PARAFAC components. Left: Scalp topographies (channel loadings) showing the spatial distribution of each component’s contribution across electrodes. Middle: Bispectral patterns (frequency loadings) of the two components in the frequency-by-frequency domain, where warm colors indicate stronger nonlinear phase coupling. Right: Bar plots illustrating the distribution of loading magnitudes for each component. **(B)** State projection of the two components at stable anesthesia stages. Group-averaged projection weights of the slow (Component 1, blue) and alpha (Component 2, orange) components across the five stable states (S1–S5). Error bars denote the standard error of the mean. **(C)** Temporal evolution of component weights across the five states. Phase-plane trajectory in the W1–W2 space, where W1 represents the weight of the slow component and W2 represents the weight of the alpha component. Each color-coded segment corresponds to one state (S1–S5), showing the dynamic shift of coupling patterns from awake to deep anesthesia.

Sliding time-window trajectory analysis quantified the dynamic transitions (Figure 5C). Across the four sequential transitions T1(S1–S2), T2(S2–S3), T3(S3–S4), T4(S4–S5), net displacements were 0.2844, 0.2166, 0.1738, and 0.0873, with directness values of 0.0731, 0.1134, 0.0524, and 0.0230, respectively. Qualitative inspection indicated that T3 and T4 became progressively restricted compared to early transitions.

To rigorously validate these observations at the single-subject level, we further calculated three dimensionless trajectory kinetic metrics for each individual participant (*N* = 17) across all four transition epochs: mean instantaneous speed, spatial diffusion and trajectory directness, which were derived from Euclidean distances of normalized PARAFAC projection weights within the W₁-W₂ phase plane. Group descriptive statistics were presented as mean ± standard error of the mean (SEM): mean instantaneous speed (T1 = 0.2808 ± 0.03377, T2 = 0.1855 ± 0.01427, T3 = 0.09597 ± 0.008082, T4 = 0.1088 ± 0.008272); spatial diffusion (T1 = 0.04208 ± 0.01350, T2 = 0.02723 ± 0.005737, T3 = 0.01132 ± 0.003470, T4 = 0.01244 ± 0.002956); trajectory directness (T1 = 0.07991 ± 0.01047, T2 = 0.06782 ± 0.008778, T3 = 0.03154 ± 0.002504, T4 = 0.03506 ± 0.004370).

Repeated-measures Friedman test revealed significant overall differences across transitions for mean instantaneous speed ( $\chi^{2}$ = 39.28, *p* < 0.0001), as well as global statistical disparities in spatial diffusion and trajectory directness. Focused pairwise comparisons targeting the critical consciousness-loss transition T2 and subsequent deep anesthesia stages T3 and T4 demonstrated consistent statistical patterns across all three metrics. For mean instantaneous speed, a highly significant difference was detected between T2 and T3 (adjusted *p* = 0.002), while no significant distinction existed between T3 and T4 (*p* > 0.05). Spatial diffusion differed significantly between T2 and T3 (adjusted *p* = 0.04), with comparable values between T3 and T4 (*p* > 0.05). Trajectory directness also exhibited a highly significant separation between T2 and T3 (adjusted *p* = 0.02), whereas T3 and T4 showed no statistically detectable divergence (*p* > 0.05). All kinetic parameters declined markedly from T2 to T3, whereas stable, confined local oscillations without prominent global shifts persisted throughout T3 and T4.

To further dissociate discrete state transitions from continuous temporal drift in neural coupling dynamics, we fitted two competing temporal models—a piecewise stepwise constant model and a fourth-order global polynomial model—to each participant’s component-weight time series. Model performance was evaluated using Akaike Information Criterion (AIC), coefficient of determination ( R2 ), and root-mean-square error (RMSE). For the prefrontal slow-wave component (W_1_), 14 out of 17 participants exhibited lower AIC values for the stepwise model, indicating individual-level preference for discrete dynamical structure. At the group level, paired t-test confirmed that the stepwise model yielded significantly smaller AIC values ( t=−5.124,p0.0001 ), together with higher explained variance ( R2=0.270vs.0.232 ) and lower fitting error (RMSE = 0.1663 vs. 0.1708) relative to the continuous polynomial model. Consistently, the mean ΔAIC(ΔAIC=AIC_stepwise−AIC_continuous) for W_1_ was −11.96 ± 9.63, demonstrating robust and consistent fitting superiority of the discrete stepwise configuration across subjects.

In contrast, the occipital alpha component (W_2_) exhibited different dynamical characteristics. Only 5 out of 17 participants favored the stepwise model, with no significant group-level AIC difference between models ( t=1.216,p=0.2416,mean ΔAIC=3.27±11.10 ). Both models achieved comparable performance ( R2=0.424vs.0.422,RMSE=0.2565vs.0.2565 ), indicating that discrete segmentation provided no fitting advantage for alpha dynamics. This component-specific dissociation indicates that fronto-prefrontal slow-wave coupling undergoes discrete, stepwise reconfiguration during propofol-induced unconsciousness, whereas posterior alpha coupling features evolve predominantly through smooth, continuous temporal drift. In summary, propofol-induced cortical bicoherence dynamics reflect a non-linear process wherein loss of consciousness manifests as a discrete state-space reconfiguration, followed by localized baseline oscillations throughout deep anesthesia.

## 4 Discussion

By integrating PARAFAC decomposition of linear TFR with nonlinear bicoherence analysis of EEG, this study provides complementary insights into the reorganization of brain dynamics during progressive propofol anesthesia. PARAFAC decomposition of TFR identified three distinct components reflecting a gradual redistribution of spectral power across anesthesia states. In contrast, bicoherence PARAFAC revealed state-dependent changes in nonlinear coupling, with a pronounced increase in slow-wave coupling during the transition from light sedation to deep anesthesia, followed by relative stabilization at higher anesthetic levels. Together, these findings indicate that progressive loss of consciousness is accompanied by both gradual spectral reorganization and distinct changes in nonlinear brain dynamics. This multidimensional framework provides a complementary approach for characterizing the evolving neural dynamics associated with loss of consciousness.

### 4.1 A unified framework of consciousness transition: from high-frequency integration to low-frequency segregation

Multiple analyses consistently indicate that propofol anesthesia leads to a simplification of brain dynamics. The phenomena of “low-frequency enhancement with high-frequency suppression” and the spectral anterior shift, as revealed by time-frequency analysis and topographical maps (Ching et al., 2010; Manohara et al., 2025), represent macroscopic manifestations of progressively suppressed brain activity during deepening anesthesia. In parallel, the spatially distributed scalp patterns of within-channel quadratic phase coupling identified by auto-bicoherence further characterize state-dependent changes in nonlinear EEG dynamics. These findings are consistent with, but do not directly demonstrate, alterations in thalamocortical or GABAergic inhibitory mechanisms. Such circuit-level changes may represent potential mechanisms underlying the observed sensor-level spectral and nonlinear reorganization during propofol anesthesia (Ching et al., 2010; Hayashi et al., 2014; Bai et al., 2019; Kreuzer et al., 2024).

Concurrently, the monotonic decrease in approximate entropy and the intrinsic dimensionality of the signal (Alkire et al., 2008; Leung et al., 2014) further depicts, from a complexity perspective (Figure 2), the gradual simplification of brain activity as anesthesia deepens. These common non-linear analysis methods collectively demonstrate that brain activity evolves towards a lower-complexity state with deepening anesthesia, these findings show that propofol anesthesia was associated with increasingly regular EEG dynamics and reduced effective sensor-level covariance dimensionality, suggesting a progressive simplification of the recorded neural dynamics during loss of consciousness.

However, the core theoretical advancement of this study lies in demonstrating that this macroscopically gradual, quantitative process is driven by an underlying discontinuous, discrete dynamic reorganization that becomes apparent only when cross-frequency phase interdependencies are unveiled. PARAFAC decomposition of linear time-frequency power initially suggests that the state transition is not a unitary process but rather a coordinated evolution of multiple neural components with distinct spatio-temporal-spectral signatures (Figure 3). Unlike coherence analysis, which only reflects linear synchronization at the same frequency (Uno et al., 2024), bicoherence is highly sensitive to QPC across frequencies, effectively capturing the reorganization of hierarchical neural coordination mechanisms. The abrupt transition observed during the State 2 to State 3 transition—characterized by a sudden dissolution of global alpha self-coupling and a highly directed phase-space migration—strongly indicates that State 3 represents a critical state-space boundary (Tononi, 2004; Oizumi et al., 2014) (Figure 4). This indicates that loss of consciousness is a nonlinear reorganization of neural ensemble coordination patterns. After crossing a critical point, brain dynamics collapse from a high-dimensional, flexible state supporting consciousness to a low-dimensional, stereotypical unconscious state.

### 4.2 Multi-dimensional extraction of linear neurodynamic profiles across graded anesthetic states

We employed PARAFAC to decompose the time-frequency power tensor, extracting three components with neurophysiological significance. Component 1 (temporo-parietal low-frequency activity) gradually intensified, reflecting the establishment of global slow-wave synchronization and marking the progressive breakdown of the brain’s information integration capacity. Component 2 (fronto-central Alpha activity) increased in a graded manner, accounting for the spectral anterior shift phenomenon, potentially originating from resonant interactions within thalamocortical circuits (Redinbaugh et al., 2020; Weiner et al., 2023). Its “rhythmic inhibition” effect may impede sensory information transmission (Zhang et al., 2023). Component 3 (occipito-parietal alpha activity), acting as a “wakefulness-related component,” rapidly diminished, signifying the disintegration of the neural substrate necessary for maintaining wakefulness. The fact that these three components evolved at different rates indicates that anesthesia results from the coordinated action of multiple discrete spatio-temporal-spectral mechanisms, providing evidence that macroscopic quantitative changes originate from microscopic discrete reorganization.

### 4.3 Bicoherence reveals nonlinear phase coupling dynamics as a window into the LOC boundary

Bicoherence analysis provided a unique perspective for revealing the nonlinear dynamics near the critical point (Dong et al., 2022). The innovative application of PARAFAC to the whole-brain bicoherence tensor enabled us to extract nonlinear coupling motifs from the complex coupling spectrum in a data-driven manner. This approach builds upon the established use of PARAFAC for decomposing multidimensional EEG data into distinct spatial, spectral, and temporal components, together with higher-order spectral analysis for characterizing nonlinear interactions in neural signals (Andersson and Bro, 2000; Miwakeichi et al., 2004). The analysis revealed that near the critical concentration for loss of consciousness (~2 mcg/ml), a specific bicoherence component (Component 1) exhibited an abrupt, step-like onset and remained sustained during deep anesthesia (Figure 5). This step-like emergence and plateau suggest a qualitative, discontinuous reorganization of cross-frequency phase coupling occurring at the threshold of unconsciousness. Based on this striking divergence between gradual linear power shifts and discrete nonlinear decoupling, we propose that state 3 serves as the neurodynamic threshold for the loss of consciousness. Furthermore, the dominant <5 Hz frontal coupling component under deep anesthesia indicates the brain is trapped in slow, stereotyped reverberating loops, with severely impaired flexibility in information processing(Mesbah-Oskui, 2015). This aligns with theoretical models suggesting impaired information integration and transfer under deep anesthesia (El-Beheiry and Puil, 1989; Flohr, 1995; Uhrig et al., 2014). Nonlinear analysis successfully captured the signal of qualitative change at the critical point, compensating for the limitations of linear analysis in describing abrupt state transitions.

Although separate statistical modeling of individual tensor projection axes reveals heterogeneous local dynamic patterns, our core conclusion centers on the macroscopic behavior of the integrated whole-brain nonlinear coupling system. Joint visualization of the two orthogonal tensor-derived projection weights confirms a discrete, irreversible critical transition at ~2 μg/mL propofol, representing a system-level reconfiguration of cortical cross-frequency coordination during the loss of consciousness.

## 5 Limitations and future directions

This study has several limitations. The sample size (*n* = 17) is relatively small, and analyzing EEG data from a greater number of patients may enhance the strength of the evidence. Data from healthy volunteers in a resting state may not fully reflect the complexity of clinical anesthesia. The lack of simultaneous neuroimaging data limits precise anatomical localization of the EEG components. The current bicoherence analysis was restricted to within-channel signals, unable to reveal nonlinear connectivity between brain regions. The precise physiological basis of the bicoherence changes requires further investigation. Future studies should integrate multimodal neuroimaging, fine-grained behavioral assessment, and whole-brain network-level analyses.

## 6 Conclusion

Our study systematically elucidates the neurodynamic mechanisms underlying graded propofol anesthesia by integrating multi-dimensional linear and nonlinear EEG features. Our results reveal that macroscopically gradual functional suppression masks discrete reconfigurations of nonlinear cortical networks. Utilizing the PARAFAC framework, we disentangled core spatio-spectral components and captured an abrupt, step-like onset of low-frequency phase coupling precisely at State 3, which persisted into deeper anesthetic stages. This trajectory signifies a sharp contraction of brain dynamics, transitioning from a flexible, high-dimensional conscious state to a rigid, low-dimensional regime. Model comparisons on isolated single tensor projection axes show that while the prefrontal slow-wave projection favors a discontinuous stepwise model, the occipital alpha projection exhibits smooth temporal drift. Importantly, because individual axes represent only partial dimensions of the high-dimensional coupling tensor, global brain dynamics cannot be defined by a single component alone. The combined two-projection phase-space trajectory consistently demonstrates a macroscopic discrete transition at the LOC threshold, confirming system-level stepwise neural reorganization under graded propofol anesthesia.

## Data Availability

The raw data supporting the conclusions of this article will be made available by the authors, without undue reservation.
