# Supplementary material for: Tensor decomposition reveals discrete cortical reorganization under graded propofol anesthesia
Source: Front Neurosci. 2026 Oct 2;20:1950745. doi: 10.3389/fnins.2026.1950745 (PMC13672537; doi:10.3389/fnins.2026.1950745)
Supplement: Supplementary file 1 [file Supplementary_File_1.docx]

Supplementary Material

# 1 Supplementary Figures and Tables

## 1.1 Supplementary Figures

Figure S1. The study CONSORT diagram.

Figure S2: Assessment of sedation depths across different states. The bar plot illustrates the Observer’s Assessment of Alertness/Sedation (OAA/S) scores across five distinct states ((State 1: awake baseline; States 2–5: target propofol effect-site concentrations of 1, 2, 3, and 4mcg/ml, respectively)) across all 17 subjects. Data are presented as mean ±SD (n = 17). Pairwise comparisons between adjacent states were performed using [Man-Whitney comparison test]. Significance levels are indicated above the brackets: ns, not significant (p > 0.05); *p < 0.05; **p < 0.01; ***p < 0.001).

Figure S3: Explained variance (A) and cumulative variance (B) of each component in PARAFAC for TFR, related to Figure 3.

Figure S4: Bicoherence of channel 1-30, from state1 to state 5, related to Figure 4.

Figure S5: Bicoherence of channel 31-62, from state1 to state 5, related to Figure 4.

## 1.2 Supplementary Tables

| Characteristics | n=17 |
| --- | --- |
| Age(yr), mean (range) | 46 (28~62) |
| Weight(kg), mean (SD) | 67.7 (11.5) |
| Height(cm), mean (SD) | 169 (7.4) |
| BMI, mean (SD) | 23.7 (2.9) |
| Sex(male), n (%) | 17 (59%) |

Table 1. Patient characteristics and variables.

| Patient  State | P_1_ | P_2_ | P_3_ | P_4_ | P_5_ | P_6_ | P_7_ | P_8_ | P_9_ | P_10_ | P_11_ | P_12_ | P_13_ | P_14_ | P_15_ | P_16_ | P_17_ |
| --- | --- | --- | --- | --- | --- | --- | --- | --- | --- | --- | --- | --- | --- | --- | --- | --- | --- |
| S1 | 5 | 5 | 5 | 5 | 5 | 5 | 5 | 5 | 5 | 5 | 5 | 5 | 5 | 5 | 5 | 5 | 5 |
| S2 | 5 | 5 | 5 | 4 | 5 | 5 | 5 | 4 | 4 | 5 | 5 | 5 | 5 | 5 | 5 | 5 | 4 |
| S3 | 4 | 4 | 4 | 3 | 2 | 2 | 3 | 2 | 2 | 3 | 4 | 4 | 2 | 2 | 2 | 2 | 2 |
| S4 | 2 | 2 | 2 | 2 | 1 | 1 | 2 | 2 | 1 | 1 | 2 | 1 | 1 | 1 | 1 | 1 | 1 |
| S5 | 1 | 1 | 1 | 1 | 1 | 1 | 1 | 1 | 1 | 1 | 1 | 1 | 1 | 1 | 1 | 1 | 1 |

Table 2. OAAS scores for each patient at different drug concentrations. S1–S5 represent propofol concentrations of 0, 1, 2, 3, and 4 mcg/ml, respectively. OAAS (Observer's Assessment of Alertness/Sedation) scores range from 0 to 5, with higher scores indicating greater alertness. A score of **5** indicates that the subject responds readily to their name spoken in a normal tone; **4** indicates a lethargic response to their name spoken in a normal tone; **3** indicates a response only after the name is called loudly or repeatedly; **2** indicates a response only after mild prodding or shaking; **1** indicates a response only after a painful trapezius squeeze; and **0** indicates no response after a painful trapezius squeeze.

|  | State1 | State2 | State3 | State4 | State5 |
| --- | --- | --- | --- | --- | --- |
| State1 | / | 0.058 | 0.072 | 0.019 | 0.005 |
| State2 | 0.058 | / | >0.99 | >0.99 | >0.99 |
| State3 | 0.072 | >0.99 | / | >0.99 | >0.99 |
| State4 | 0.019 | >0.99 | >0.99 | / | >0.99 |
| State5 | 0.005 | >0.99 | >0.99 | >0.99 | / |

Table 3. Diagonal bicoherence in the prefrontal across states.

Statistical comparison of normalized diagonal bicoherence in the prefrontal across states. One-way ANOVA with FDR correction; p-values are shown in the table (significance threshold: p < 0.05).

|  | State1 | State2 | State3 | State4 | State5 |
| --- | --- | --- | --- | --- | --- |
| State1 | / | 0.047 | 0.089 | 0.0069 | 0.001 |
| State2 | 0.047 | / | >0.99 | >0.99 | >0.99 |
| State3 | 0.089 | >0.99 | / | >0.99 | >0.99 |
| State4 | 0.0069 | >0.99 | >0.99 | / | >0.99 |
| State5 | 0.001 | >0.99 | >0.99 | >0.99 | / |

Table 4. Diagonal bicoherence in the frontal across states.

Statistical comparison of normalized diagonal bicoherence in the frontal area across states. One-way ANOVA with FDR correction; p-values are shown in the table (significance threshold: p < 0.05).

|  | State1 | State2 | State3 | State4 | State5 |
| --- | --- | --- | --- | --- | --- |
| State1 | / | 0.001 | 0.037 | 0.001 | 0.001 |
| State2 | 0.001 | / | >0.99 | >0.99 | >0.99 |
| State3 | 0.037 | >0.99 | / | >0.99 | >0.477 |
| State4 | 0.001 | >0.99 | >0.99 | / | >0.99 |
| State5 | 0.001 | >0.99 | >0.99 | 0.477 | / |

Table 5. Diagonal bicoherence in the central across states.

Statistical comparison of normalized diagonal bicoherence in the central area across states. One-way ANOVA with FDR correction; p-values are shown in the table (significance threshold: p < 0.05).

|  | State1 | State2 | State3 | State4 | State5 |
| --- | --- | --- | --- | --- | --- |
| State1 | / | 0.162 | 0.018 | 0.001 | 0.001 |
| State2 | 0.162 | / | >0.99 | 0.037 | 0.002 |
| State3 | 0.018 | >0.99 | / | 0.28 | 0.024 |
| State4 | 0.001 | 0.037 | 0.28 | / | >0.99 |
| State5 | 0.001 | 0.002 | 0.024 | 0.477 | / |

Table 6. Diagonal bicoherence in the parietal across states.

Statistical comparison of normalized diagonal bicoherence in the parietal across states. One-way ANOVA with FDR correction; p-values are shown in the table (significance threshold: p < 0.05).

|  | State1 | State2 | State3 | State4 | State5 |
| --- | --- | --- | --- | --- | --- |
| State1 | / | 0.196 | 0.002 | <0.001 | <0.001 |
| State2 | 0.196 | / | >0.99 | >0.99 | 0.339 |
| State3 | 0.002 | >0.99 | / | 0.072 | >0.99 |
| State4 | <0.001 | <0.001 | 0.072 | / | >0.99 |
| State5 | <0.001 | 0.339 | >0.99 | >0.99 | / |

Table 7. Diagonal bicoherence in the occipital across states.

Statistical comparison of normalized diagonal bicoherence in the occipital across states. One-way ANOVA with FDR correction; p-values are shown in the table (significance threshold: p < 0.05).

|  | State1 | State2 | State3 | State4 | State5 |
| --- | --- | --- | --- | --- | --- |
| State1 | / | 0.109 | 0.037 | >0.99 | >0.99 |
| State2 | 0.109 | / | 0.072 | 0.005 | 0.562 |
| State3 | 0.037 | 0.072 | / | >0.99 | >0.99 |
| State4 | >0.99 | 0.005 | >0.99 | / | >0.99 |
| State5 | >0.99 | 0.562 | >0.99 | >0.99 | / |

Table 8. Diagonal bicoherence in the temporal across states.

Statistical comparison of normalized diagonal bicoherence in the temporal across states. One-way ANOVA with FDR correction; p-values are shown in the table (significance threshold: p < 0.05).

# 2 Supplementary Methods

1. Software Environment and Version Standardization

To prevent algorithmic discrepancies, parameter shifts, and reproducibility issues arising from software version iterations, the complete electroencephalographic (EEG) analytical pipeline in this study was implemented using strictly fixed software and toolbox versions. All numerical computations, tensor decompositions, and statistical procedures were executed within a unified hardware and software computing environment.

**1.Core Platforms and Third-Party Toolboxes**

- **Primary Computing Platform:** The core analytical platform was **MATLAB R2020a** (MathWorks, Natick, MA, USA). All underlying numerical calculations, matrix operations, iterative optimizations, and statistical aggregations relied on its native kernel, ensuring fixed floating-point arithmetic rules, convergence criteria, and computational precision.
- **EEG Preprocessing Pipeline:** Raw EEG data importing, re-referencing, independent component analysis (ICA), and artifact identification/rejection were conducted using the stable **EEGLAB (v2023.0)** release. Locking this toolbox version avoided systematic errors introduced by version-dependent ICA optimization routines, filtering algorithms, or artifact classification thresholds.
- **Spectral and Time-Frequency Analysis:** Time-domain segmentation, overlapping epoch reconstruction, multi-window time-frequency decomposition, and array reshapes were performed using the archived **FieldTrip toolbox (v20230118)**. This specific release provided a standardized, field-tested environment for time-frequency calculations, taper windowing, and array normalization routines.
- **Multi-Way Tensor Decomposition:** Multi-dimensional tensor decomposition, Alternating Least Squares (ALS) iterative optimization, non-negativity constraint solving, Core Consistency Diagnostic (CORCONDIA) computation, and multi-run random initializations relied entirely on the **N-way Toolbox (v3.30)**. As the gold-standard framework for PARAFAC tensor modeling in neuroscience, its ALS step updates, residual sum-of-squares formulas, and constraint solvers were utilized in their standard implementation without custom code alterations.

**2.Custom Algorithms and Statistical Software**

- **Approximate Entropy (ApEn):** Quantifications of temporal dynamic complexity (ApEn) were executed using custom scripts strictly implementing Pincus's canonical formulation (1991), operating without third-party dependencies to guarantee pure, fully traceable time-series dynamic modeling.
- **Custom Analytical Pipelines:** Bicoherence calculations, trial-level hierarchical filtering, tensor dimension permutations, modal loading alignments, least-squares weight projections, and phase-space trajectory reconstructions were executed using fully customized, in-house MATLAB functions. All threshold parameters, iteration bounds, convergence tolerances, and constraint vectors were explicitly defined without implicit parameters, guaranteeing 100% workflow reproducibility.
- **Statistical Inference and Visualization:** All downstream inferential hypothesis testing, post-hoc corrections, and data visualizations were executed in **GraphPad Prism (v8.0)**, ensuring uniform execution of nonparametric statistical tests, multiple comparison adjustments, and graphical rendering across all experimental endpoints.

2. Raw EEG Preprocessing, Filtering, ICA Artifact Correction, and Re-referencing Protocols

Data were analyzed from 17 healthy participants undergoing general anesthesia. All participants were processed using an identical, fully standardized preprocessing pipeline without individualized parameter tuning to strictly eliminate human operator bias. Raw electroencephalographic (EEG) recordings collected via the Curry neuroacquisition system were imported while preserving only functional scalp channels and anesthesia event markers. Automatic electrode template matching was disabled to prevent coordinate mapping distortions. Four auxiliary electrooculogram (EOG) channels (HEOG, VEOG, HEO, and VEO) were removed early in preprocessing, retaining solely the 62 standard scalp electrodes based on the international 10–20 system for downstream neural feature analyses. Raw signals were uniformly downsampled to 500 Hz, preserving fine temporal details across delta to high-gamma oscillations while reducing data redundancy and computational overhead during sliding-window segmentation.

**1.Two-Stage Zero-Phase Filtering**

Denoising was performed using a two-stage zero-phase finite impulse response (FIR) linear filtering scheme, ensuring zero phase distortion across the time series:

- Broadband Passband Filtering: A 0.5–80 Hz bandpass filter was applied to remove low-frequency DC baseline drifts, slow respiratory artifacts, and high-frequency electromyographic (EMG) or equipment noise above 80 Hz.
- Narrowband Notch Filtering: A 48–52 Hz notch filter was implemented to selectively attenuate 50 Hz power-line interference. The ultra-narrow stopband minimized power attenuation in adjacent alpha and beta oscillatory bands, achieving an optimal trade-off between noise suppression and signal preservation.

**2. ICA Decomposition, Deep Learning Component Classification, and Reconstruction**

Following time-domain filtering, extended Infomax adaptive Independent Component Analysis (ICA) was executed to unmix neural source signals from non-linear artifactual sources. Independent components (ICs) were automatically classified using the EEGLAB-integrated ICLabel deep learning framework. A strict confidence threshold of 0.9 was enforced: components were designated as artifacts and subtracted if and only if the predicted probability for muscle, eye, heart, line noise, channel noise, or equipment artifacts was $\geq$0.9. All neural ICs were retained without threshold restrictions to maximize the preservation of true physiological dynamics and prevent over-cleansing. Artifact correction was executed purely via component subtraction without waveform interpolation, spatial smoothing, or data imputation, maintaining original signal fidelity and amplitude validity.

**3. Average Mastoid Re-referencing and Dual-Segmentation Pipelines**

Following ICA-based artifact subtraction, all 62 scalp channels were re-referenced to the algebraic average of the bilateral mastoids (M1 and M2). Mastoid channels were retained throughout preprocessing to establish a homogenous reference baseline across all 17 participants, eliminating inter-subject and cross-state baseline offsets.

The clean, continuous EEG time series were subsequently split into two independent epoching pipelines:

- 3-Second Epochs: Short, overlapping 3-second segments optimized for time-frequency representation (TFR) analysis across rapid anesthetic transitions.
- 7-Second Epochs: Long, overlapping 7-second segments optimized for robust, non-linear phase-phase coupling (bicoherence) computations.

Both epoching pipelines underwent independent quality control screening and feature extraction downstream.

3. Hierarchical Artifact Screening, Outlier Rejection, and Trial Retention Protocols for 7-Second Bicoherence Epochs

**1. Rationale and Segmentation Strategy for Bicoherence Analysis**

Wavelet bicoherence computations are highly sensitive to signal instability, phase discontinuity, and transient noise. Uncorrected high-amplitude artifacts, baseline drifts, or random fluctuations can directly induce spurious coupling peaks, distort bispectral matrices, and destabilize downstream tensor decompositions. To guarantee pristine data quality, a three-tier progressive hierarchical quality control framework was established specifically for the bicoherence analytical pipeline. All 17 participants and 5 anesthetic states were subjected to identical, fully standardized screening rules without individualized parameter tuning. A 7-second sliding window with a 50% temporal overlap was implemented, ensuring continuous temporal coverage while providing an extended time window necessary to optimize low-frequency phase-coupling estimations.

**2. Three-Tier Hierarchical Quality Control Pipeline**

- **1: Channel-Wise 3**$\boldsymbol{\sigma}$ **Outlier Rejection:** Within each single participant and discrete anesthetic state, the global mean ($\boldsymbol{\mu}$) and standard deviation ($\boldsymbol{\sigma}$) of the raw time series were computed independently for every channel across all available trials. Any single trial exhibiting instantaneous signal amplitudes exceeding the threshold of $\boldsymbol{[\mu\pm\sigma]}$ in any channel was immediately flagged as a high-amplitude outlier trial and purged. This step eliminated severe ocular movements, gross body motion, and transient electrode displacement spikes.
- **2: Global Noise Quantification Scoring:** For all remaining valid trials, signals across all 62 channels were Min-Max normalized to remove baseline amplitude variations. The mean normalized fluctuation amplitude across all channels was then computed for each trial to yield a scalar global noise score. Higher noise scores reflected greater signal turbulence and a lower proportion of phase-consistent physiological neural activity.
- **3: Fixed-Ratio Stratified Selection:** A strict, pre-specified 50% trial rejection ratio was enforced across all conditions. All 3$\boldsymbol{\sigma}$ outlier trials identified in Tier 1 were prioritized for removal. If the number of $\boldsymbol{3}\boldsymbol{\sigma}$ outliers was fewer than 50% of the total trial pool, additional trials were systematically rejected in descending order of their Tier 2 noise scores until exactly 50% of the cleanest, highest phase-stability trials were retained for bicoherence computation.

**3. Auditability and Data Provenance**

All trial rejection flags, individual noise scores, and exact counts of discarded versus retained epochs were fully logged and archived. The complete quality control workflow is 100% traceable and reproducible. These screened, high-quality 7-second epochs served as the sole valid data input for subsequent bicoherence matrix calculations and multi-way tensor modeling.

4. Low-Variance Trial Selection and Sample Standardization Protocols for 3-Second TFR Epochs

**Rationale and Short-Epoch Segmentation Strategy**

To track rapid, dynamic transitions across anesthetic states, linear time-frequency representation (TFR) oscillatory analysis was conducted using short 3-second epoch segments. This window length achieved an optimal trade-off between temporal dynamic resolution and spectral estimation stability. In parallel, a rigorous trial quality control system was established to eliminate potential biases in PARAFAC tensor decomposition loadings caused by trial quality heterogeneity or unequal sample sizes across experimental conditions.

**1. Global Variance Optimization and Sample Size Standardization**

Following the initial removal of 3$\sigma$ transient artifact trials, a full-dimensional global variance optimization strategy was implemented to standardize sample sizes across all participants and anesthetic states, targeting a fixed quota of the 150 most stable trials per anesthetic state. The selection algorithm proceeded as follows:

- **Spectral Matrix Flattening:** For each candidate 3-second trial, the complete power spectral matrix across all 62 scalp channels and 89 discrete frequency bins (0.5-50Hz) was extracted and flattened into a single high-dimensional feature vector.
- **Global Variance Quantification:** The global variance across this flattened vector was computed as a direct proxy for overall oscillatory stability. Lower variance indicated higher spatio-spectral power uniformity, minimal random noise perturbation, and superior signal-to-noise ratio of pure physiological neural oscillations.
- **Ascending Rank Ordering and Retention:** All valid trials within a given participant and anesthetic state were ranked in ascending order of their global variance scores. The top 150 trials exhibiting the lowest global variance were retained. If an isolated participant/state condition contained fewer than 150 total valid trials post-denoising, all available valid trials were retained without artificial truncation to preserve maximum data integrity.

**2. Transparency, Traceability, and Tensor Input Quality**

Individual trial variance metrics, overall variance distribution ranges per anesthetic state, extreme value parameters, and selected trial indices were logged and archived at every step. This ensured complete transparency and end-to-end data traceability. These standardized, low-variance trials served as the clean data input for multi-window time-frequency decomposition and subsequent group-level multi-way tensor construction, guaranteeing that input features to the PARAFAC model were free from quality stratification and sample size biases.

5. Multi-Window Time-Frequency Decomposition, Dimensional Reconstruction, Temporal Alignment, and Five-Dimensional Tensor Normalization Protocols

**1. Multi-Window Time-Frequency Feature Extraction**

Time-frequency representation (TFR) power spectral feature extraction was conducted using the multi-taper fast Fourier transform (MTMFFT) algorithm implemented in the FieldTrip toolbox via the ft_freqanalysis function. Compared to conventional short-time Fourier transforms, this multi-window approach offers markedly lower spectral variance and superior stability, making it ideally suited for resolving subtle, low-amplitude neural oscillatory signals. A Hanning taper was applied uniformly across all epoch segments. The frequency analysis range was locked between 0.5 and 50 Hz, fully covering all classical electroencephalographic (EEG) oscillatory bands (delta, theta, alpha, beta, and gamma) with 89 discrete, finely sampled frequency bins to guarantee sufficient spectral resolution. The decomposition preserved raw power spectra at the single-trial level without trial averaging or artificial compression of single-trial dynamical variance, thereby retaining optimal sample dimensionality for downstream multi-dimensional tensor feature learning.

**2. Dimensional Reconstruction and Temporal Alignment**

The raw output array of the TFR analysis was initially structured as [Trial $\times$Channel $\times$Frequency]. To accommodate the three-way PARAFAC tensor modeling framework, standardized dimensional permutation and temporal alignment were performed: using the mid-point of each 3-second epoch segment as a canonical time stamp, a continuous pseudo-timeline was constructed to align discrete epoch segments across time. Subsequently, the data array was permuted into a standardized [Channel $\times$ Frequency$\times$ Trial/Time] structure, establishing unified alignment across spatial, spectral, and temporal modes.

**3. Five-Dimensional Array Assembly and Strict Within-Subject Normalization Protocols**

Following dimensional reconstruction, a raw five-dimensional array structured as [Participant $\times$ Channel $\times$ Frequency $\times$ Trial $\times$ Anesthetic State] was generated. To completely prevent cross-participant and cross-state data leakage or feature contamination caused by global normalization after tensor concatenation, a strict protocol of "independent within-subject/state normalization prior to group-level tensor concatenation" was implemented.

Normalization was executed strictly within each isolated single-participant, single-state dataset using a Min-Max scaling formulation:

$X_{norm}=\frac{X-min(X)}{max(X)-min(X)+\varepsilon}$，where $\varepsilon={10}^{-8}$ represents a tiny offset constant added to prevent numerical division-by-zero errors in steady-state, flatline signals.

This procedure independently scaled the spectral power values of each individual participant within each discrete state to a bounded [0, 1] interval, completely eliminating systemic biases arising from individual scalp impedance, baseline power variations, and global energy shifts across anesthetic depth levels. Consequently, the tensor model was forced to extract solely relative neural features—specifically spatial topologies, spectral shapes, and temporal evolutions—rather than absolute magnitude variations. Only after all independent single-participant/state normalizations were completed were the datasets concatenated to construct the final group three-way tensor for PARAFAC decomposition, guaranteeing absolute group independence, comparability, and complete protection against cross-condition feature leakage.

6. 100-Run Random Initialization for Global Optimality Verification: Mitigating Local Minima Concerns

**1. Rationale and Random Initialization Framework**

Because Alternating Least Squares (ALS) optimization in PARAFAC tensor modeling carries an inherent risk of initialization sensitivity—where arbitrary initial loading matrices can lead to sub-optimal local minima—a 100-run unfiltered, fully retained independent random initialization scheme was implemented. This rigorously verified that the reported decomposition represented the unique, globally stable optimal solution, free from selection bias or selective reporting. The complete 17-participant tensor decomposition served as the baseline reference solution (refFac, corresponding to the standard canonical modes in the main text). Across 100 independent iterations, all hyperparameters, non-negativity constraints, and tensor ranks were held constant, while initializing each run with newly generated random starting matrices—devoid of prior warm-starts, inherited baseline parameters, or manual post-selection filtering.

**2. Convergence Audit and Alignment Pipeline**

For every run, key optimization diagnostics were systematically recorded, including sum of squared errors (SSE) residuals, iteration counts, Core Consistency Diagnostic (CORCONDIA) values, and final convergence statuses. All 100 independent fitting runs achieved successful numerical convergence with zero failures, zero exclusions, and zero anomalous cases. To resolve intrinsic multilinear ambiguities associated with component order permutations and sign flips, all converged factor matrices were automatically aligned and sign-corrected relative to refFac using the parafac_align algorithm. Pearson correlation coefficients were subsequently computed for each mode and component against the baseline reference solution.

**3. Global Convergence and Model Stability Outcomes**

Across all modes, the median correlation coefficients for the primary physiologically meaningful Components 1 and 2 exceeded **0.97**, with the vast majority of modal correlations approaching **1.0**. These findings demonstrate that arbitrary random initializations consistently converge to the identical global optimum. The tensor model exhibits no local minima

7. Leave-One-Out (LOO) Cross-Validation: Mitigating Individual Outlier Dominance

**1. Rationale and Cross-Validation Framework**

To exclude the possibility that overall tensor decomposition outcomes were driven or distorted by single-subject physiological anomalies, idiosyncratic noise, or individual baseline biases, a 17-fold Leave-One-Out (LOO) cross-validation procedure was conducted. Iteratively across 17 rounds, one participant ($N = 1, 2,\boldsymbol{\cdots}17$) was omitted from the dataset. The remaining 16-participant sub-cohorts independently underwent the complete analytical pipeline, including within-subject Min-Max scaling, five-dimensional array reconstruction, and PARAFAC tensor decomposition. All hyperparameters, iteration limits, and non-negativity constraints were maintained identically to those of the full-cohort baseline model.

**2. Modal Alignment and Stability Metrics**

In each cross-validation iteration, the resulting decomposed spatial, spectral, and temporal mode loadings were aligned and matched against the canonical full-cohort baseline factor loadings (refFac). For every round, Pearson correlation coefficients between the sub-cohort and baseline loading vectors were computed across all modes, alongside tracking the Core Consistency Diagnostic (CORCONDIA) values.

**3. Cross-Validation Outcomes and Generalizability**

Across all 17 iterations, the sub-cohort decomposition modes exhibited exceptional convergence with the global baseline model. Loading correlation coefficients consistently maintained high values (r$\boldsymbol{>}$0.95), and the core consistency index displayed no significant degradation following single-subject omissions. These results confirm that the spatial, spectral, and temporal coupling patterns extracted in this study represent robust, generalized population-level neural signatures rather than artifacts of individual outliers, demonstrating high model generalizability and statistical reliability across the cohort.

8. Sensitivity Validation of Trial Selection Thresholds

To address potential concerns regarding trial-sampling bias arising from the fixed selection of 150 low-variance trials for the TFR analysis and 50% of the lowest-noise trials for the bicoherence analysis, we additionally performed a sensitivity analysis across multiple trial-selection thresholds. This analysis was designed to exclude potential bias arising from preferential trial selection or subjective threshold determination.

The numbers of retained trials and exclusion proportions were not arbitrarily selected. The 150-trial criterion represented the maximum stable number of valid trials that could be uniformly matched across all animals and anesthesia states, thereby standardizing the dimensionality of the datasets and minimizing bias caused by unequal trial numbers. For the bicoherence analysis, the 50% exclusion criterion was selected to achieve a balance between noise removal and signal preservation, allowing effective removal of transient artifacts while retaining sufficient physiologically meaningful coupling signals.

To further assess the robustness of these selection criteria, we performed permutation-based sensitivity analyses using a range of alternative thresholds, including exclusion proportions of 30%, 40%, 60%, and 70%, as well as alternative retained-trial numbers of 120 and 180. Across all tested thresholds, the core spatiotemporal and spectral component loadings remained highly consistent with the reference solutions, with correlation coefficients consistently exceeding 0.95. Moreover, no significant differences were observed in the core decomposition patterns, physiological characteristics, or statistical relationships of the models.

These results demonstrate that the main findings were robust to variations in trial-selection thresholds and were not dependent on a specific, arbitrarily defined criterion. Collectively, the three complementary validation procedures—initialization perturbation, individual-outlier assessment, and trial-sampling threshold sensitivity analysis—provided comprehensive validation of the uniqueness, stability, and robustness of both PARAFAC model solutions and demonstrated that the principal findings were reproducible across alternative analytical specifications.

9. Construction Principles of Standardized Spatio-Spectral Coupling Templates

Based on the stable baseline PARAFAC decomposition, the spatial loading matrix $A(62 channels\times3components)$ and the frequency loading matrix $C(50frequency bins\times3components)$were extracted. The first two core components, which possessed explicit physiological interpretations, were selected to construct standardized, fixed spatio-spectral coupling templates via vector outer products.

The canonical templates were computed using the outer product formulation:

$\boldsymbol{T}_{\mathbf{1}}=\boldsymbol{A}_{\mathbf{1}}\otimes\boldsymbol{C}_{\mathbf{1}}^{T},\boldsymbol{T}_{\mathbf{2}}=\boldsymbol{A}_{\mathbf{2}}\otimes\boldsymbol{C}_{\mathbf{2}}^{T}$，correspond to the canonical spatio-spectral modal bases for slow-wave coupling and alpha coupling, respectively. Each template is formatted to a standardized dimension of $62channels\times50fequency bins$. Serving as fixed basis matrices, they were applied uniformly across all trials and anesthetic states for standardized trial-level fitting, remaining entirely constant throughout all analytical procedures.

10. Piecewise Stepwise vs. 4th-Order Continuous Polynomial Model Fitting, Corrected AIC Computation, and Individual-Level Model Comparison Protocols

To statistically dissociate discrete stepwise state transitions from smooth continuous neural drift at the individual level, two competitive temporal models were independently fitted to each participant’s full-length weight time series for both the slow-wave component (W_1_) and alpha component (W_2_). The total time series contained 276 trials, segmented into nine task-aligned stages with fixed boundaries corresponding to the sequence S1–T1–S2–T2–S3–T3–S4–T4–S5. The segmentation breakpoints were determined by cumulative trial lengths defined as$segLen = [28, 41, 28, 41, 28, 41, 28, 41, 28]$. Detailed model construction, fitting procedures, corrected quantitative metrics, and individual-level comparison criteria are specified below.

**1. Temporal Model Construction Rules**

- **Piecewise Stepwise Constant Model:** The stepwise model was constructed using nine binary indicator basis functions corresponding to the nine predefined task stages. Each basis function was assigned a value of 1 within its corresponding stage and 0 elsewhere. Ordinary least-squares regression was performed to estimate nine segment-wise constant amplitude parameters, allowing unconstrained instantaneous jumps at stage boundaries. The total number of free parameters for the stepwise model was $k=9$.
- **Fourth-Order Global Continuous Polynomial Model:** To avoid numerical ill-conditioning in high-order polynomial regression, raw trial time indices $t=1,2,\cdots,TotalLen$ were standardized via mean centering and z-score normalization: $t_{\text{scaled}}=(t-\overline{t})/\text{std}(t)$. A fourth-order polynomial basis $[1,t_{\text{scaled}},t_{\text{scaled}}^{2},t_{\text{scaled}}^{3},t_{\text{scaled}}^{4}]$ was constructed to capture smooth, global, nonlinear temporal evolution without discrete breakpoints or jump terms. The continuous polynomial model contained $k=5$ free parameters (including the intercept).

**2. Degree-of-Freedom Corrected Information Criteria**

To achieve unbiased and rigorous model comparison, degree-of-freedom corrected Akaike Information Criterion (AIC) and Bayesian Information Criterion (BIC) formulations were adopted, based on unbiased residual variance estimation. Let SSE denote the sum of squared residuals, N = 276 the total number of trial time points, and $k$ the number of model free parameters$(k=9 for stepwise;k=5 for polynomial)$. The unbiased residual variance was calculated as:

$$\sigma_{unbiased}^{2}=\frac{SSE}{N-\kappa}$$

The corrected information criteria were defined as:

$$AIC = N \cdot ln(\sigma_{unbiased}^{2}) + 2k$$

$$BIC= N \cdot ln(\sigma_{unbiased}^{2}) + k\cdot ln(N)$$

Additional fitting quality indicators included the coefficient of determination ($R^{2}$) and root-mean-square error (RMSE):

$$R^{2} = 1 -\frac{SSE}{SST}$$

$$RMSE = \sqrt{\frac{SSE}{N}}$$

where SST represents the total sum of squares.

**3. Individual-Level Model Selection Criteria**

Model fitting was performed independently for each participant and each component $(W_{1} slow-wave coupling,W_{2} alpha coupling)$. A binary model-preference label was defined as$Label = Stepwise if AICstep<{AIC}_{poly}$, and $Label=Polynomial$ otherwise. To quantitatively characterize individual model discrepancy, $\Delta AIC$ was computed as:

$$\Delta AIC ={AICs}_{step}- {AIC}_{poly}$$

Negative $\Delta AIC$ values indicate superior fitting performance of the discrete stepwise model, whereas positive values indicate better fit for the continuous polynomial model.

**4. Group-Level Statistical Standards**

All model fitting indices ($AIC, BIC, R^{2}, RMSE$, $\Delta AIC$) were first aggregated at the individual participant level. Only the 17 independent participant-level scalar values were used for group-level statistical inference to eliminate trial-based pseudoreplication. Paired two-tailed Student’s t-tests were applied to compare AIC values between the two competing models across participants. The statistical significance threshold was set at P < 0.05, and all t-statistics, degrees of freedom, and exact P-values are fully reported. Group-level $\Delta AIC$ results are presented as$mean \pm standard deviation (SD)$. All individual fitting results across participants and components are exported into standardized supplementary tables for full data transparency and reproducibility.

**5. Numerical Computing Environment and Reproducibility Constraints**

All model fitting procedures were implemented in MATLAB R2020a using native least-squares solvers, with no third-party toolbox dependencies. Time-series standardization, model regression, and metric calculations were performed via custom-written scripts with fully hard-coded hyperparameters, including polynomial order (n = 4), fixed segment lengths, predefined stage breakpoints, and convergence criteria. No subjective parameter tuning or adaptive optimization was applied across participants, ensuring fully homogeneous, traceable, and reproducible model fitting across all subjects.

11. Statistical Independent Sampling Units, Data Aggregation Rules, Degrees of Freedom, and GraphPad Statistical Standards

1. **Explicit Definition of Independent Sampling Units**

The sole independent statistical sampling unit in this study is defined strictly as the 17 individual human participants. All epoch time-domain segments, overlapping sliding windows, 62 scalp electrode channels, 50 frequency sampling points, 6 regional groupings, 6 oscillatory frequency bands, and multiple repeated trials represent nested within-subject repeated measurements. They possess no independent statistical status and are excluded from all degrees-of-freedom computations, thereby thoroughly resolving the concerns raised by the referee regarding pseudoreplication and artificially inflated degrees of freedom caused by overlapping segments and multi-dimensional repeated sampling. All raw, fine-grained trial-level data are reserved exclusively for tensor decomposition, dynamic model training, signal visualization, and stability validation, and are strictly excluded from direct input into inferential statistical testing.

2. **Standardized Hierarchical Mean Aggregation Rule**

All electrophysiological metrics (time-frequency power, bicoherence coupling strength, PARAFAC projection weights, dynamical trajectory metrics, and Approximate Entropy [ApEn]) undergo a uniform intra-subject mean compression pipeline. Specifically, within each individual participant and each discrete anesthetic state, arithmetic means are calculated across all valid overlapping epochs, trials, and channel subsets. This collapses fine-grained data into a four-dimensional individual mean matrix structured as [Participant $\times$Anesthetic State$\times$Brain Region$\times$Frequency Band]. Only these 17 sets of independent individual mean scalar outputs are imported into inferential statistical models, completely eliminating pseudoreplication bias.

3. **Nonparametric Statistical Framework**

All inferential hypothesis testing in this study is conducted exclusively on the participant-level aggregated mean data (N = 17), completely abandoning trial-level nested data to eliminate pseudoreplication and degrees-of-freedom inflation. A fully participant-level nonparametric statistical architecture is implemented. The core inferential procedure utilizes the Wilcoxon signed-rank paired test, which strictly accounts for the paired, within-subject repeated-measures design across the 17 independent participants, with no parametric Analysis of Variance (ANOVA) routines applied. All degrees of freedom and statistical contrasts are anchored strictly to the independent participant sample size (N = 17). The statistical sampling units are fully independent, free of data nesting or dimensional leakage, guaranteeing mathematically unbiased statistical inferences.

4. **Data Export and Software Execution Standards**

Following hierarchical mean aggregation in MATLAB, only non-nested participant-level aggregated scalar metrics (N = 17) are exported. These structured datasets are formatted into standard long-format tables and imported into GraphPad Prism (v8.0) to execute final hypothesis testing and statistical rendering, ensuring uniform algorithmic execution. Pairwise comparisons across anesthetic states and regional conditions are evaluated using the Wilcoxon signed-rank paired test, with strict Bonferroni correction applied for all multiple parallel comparisons. Statistical significance is established at a Bonferroni-adjusted threshold of p < 0.05. The entire analytical pipeline rigorously adheres to the standard EEG processing paradigm—"noise-cleared trial selection - intra-subject aggregation - participant-level statistical inference"—ensuring complete methodological self-consistency, transparency, and reproducibility.
